# Treatment of Dental Caries When Complicated with an Irritable or Exposed Pulp—Concluded

**Published:** 1856-06

**Authors:** James Taylor


### TREATMENT OF DENTAL CARIES WHEN COMPLICATED WITH AN IRRITABLE OR EXPOSED PULP---CONCLUDED.

BY JAMES TAYLOR.

In our last article on this subject, which was published in the January number of the Register, we particularly confined our remarks to that condition which immediately precedes exposure of the pulp.

We would here remark, that to avoid lengthening these articles unnecessarily, we have confined ourself to our general practice, and hence have not referred specially to what has been written by others on this subject. We do this the more freely, because we do not claim anything in our practice as original or different from what may be pursued by many others. We have tried, however, to simplify the subject and have aimed to present it in such a form as to benefit the young practitioner.

We so often meet with teeth which have been grumbling and troublesome for weeks, and sometimes months, and which have been condemned by young practitioners, that we were somewhat careful in our last to put them on their guard and not consider the nerve exposed unless they have positive evidence of it.

We come now, however, to that condition of the disease when the pulp is really exposed, and first speak of the treatment to preserve the nerve alive, and second to preserve the tooth by destroying the pulp.

When on excavating the carious portion of a tooth, we completely uncover and expose the pulp, we at once decide on the practicability of preserving it, by reference to the general irritability of the dental organs, the constitutional temperament of the patient, and whether the tooth has already given trouble.

If the general irritability is such, as to be materially affected by extremes of heat or cold, and the constitutional temperament, such as generally tend in such cases to the formation of alveolar abscess, and the teeth has shown symptoms of disquietude, we regard it as an unfavorable case.

Every dentist of observation, must have noticed that there are a class of patients, who on all occasions, where the disease has reached the nerve, are having swollen faces and abscess. This may indeed be called a constitutional tendency. There is another class of patients whose teeth decay and break away, and who yet scarcely ever complain of *tooth-ache.*

The former we generally find of a nervous temperament and strumous habit of body. The nervous system is too high strung to bear the least excitation, and the muscular tissue not energetic enough to repel the attacks of disease, it needs a repelling and recuperative power.

The latter generally possess a firm constitutional habit of body. The sanguinous and sanguino-billious. The powers of the system are well balanced, and if the nerves do become excited, the general energy is such as to give them room for play, and then restrain them in proper bounds thereafter. The same operation in one will prove successful, while in the other it will fail.

When the pulp is perfectly healthy, no special preparatory treatment is necessary, and when diseased the chance for success is very much lessened. True, it may be in an irritable condition and this irritation subdued, yet inflammatory action is more apt to be brought on from slight causes thereafter.

If the case is a favorable one, such as we have described, and in excavating the decay, the pulp organ has not been wounded, we proceed with the operation at once. The first thing to be done is to make an easy opening into the cavity, plenty of room should be procured for perfect manipulation. The carious portion should be thoroughly removed, and we prefer marking out on the base of the cavity, with the excavator, a perfect floor or basement to receive our cap. The cap is then made of gold plate and adjusted to the place formed for its reception. We then heat it over a spirit lamp and cover the surface, which goes next to the pulp, with a thin layer of Hill's soft filling. The cavity is then washed out with tepid water, sometimes first with a little creosote or spirits of camphor. Then perfectly dried and the cap adjusted, after which blocks of foil, or cylinders as they are now called, are adjusted around the walls of the cavity with their ends resting on and confining the edge of the cap to its place. We desire and aim to have this cap leave as little space between it and the pulp as possible, without any pressure on the nerve. A space here which must be filled with atmospheric air, or secretions from the pulp organ, we consider pernicious and a cause of many failures, for they are irritants.

It will be recollected that we are giving our usual practice, and that which has been as successful in our hands as any other. In some small and superficial cavities, we make our caps of a few folds of gold foil, and cover its lower surface with Hill's soft filling as in the former case. Oil silk may be used in the same way. Bees-wax, which is a non-conductor, may also be used in the same manner. These non-conductory materials are used merely as preventives of after trouble, yet we do not think they are always absolutely necessary. If cold or heat has not been a source of annoyance previous, or at the time of the operation, they will not often be afterwards.

Of late we but seldom attempt to cap and preserve the pulp alive after its exposure, unless in the front teeth, or when we have the favorable circumstances to which we have alluded. In the front teeth we prefer the preservation of the pulp with particular reference to the color of the teeth. In decay on the approximal surfaces of the front teeth, we often have an exposure as it were of the side of the pulp. The integrity of the pulp organ has been but little impaired. When this is the case its preservation is essentially important, for its circulation through the healthy dentine, may still be maintained and the tooth retain all its living characteristics. This is a condition of things in which we would institute remedial treatment if necessary to prepare such teeth for filling.

The pulp may be thus exposed to the effects of caries, and have shown considerable symptoms of irritation, such as pain on the application of heat or cold, pain from pressure of foreign substances, uneasiness from exposure, &c., &c., and still be restored to health and preserved without much trouble.

The first indication here is to remove the carious portion, for this is the direct source of irritation. After this is accomplished, the cavity should be washed out with tepid water. If the blood vessels appear to be engorged with blood, a slight scratch causing a little hemorrhage, will often be of service. This can be checked with an application of creosote or spirits of camphor. We prefer the former, generally, because of its antiseptic property, and from the fact that it relieves the pain instead of increases it. We more frequently apply in such cases creosote and tannin on a pledget of cotton, let it remain while we fill perhaps one or two cavities, then remove our application dry out the cavity and fill. In other cases when we have much irritation, we allow the creosote and tannin to remain twenty-four hours, and if the irritation is not completely subdued reapply. In cases, when admissible, a lead cap may be adjusted over the pulp and the cavity dried out and fill with adhesive wax. Care should be taken that pressure is not made on the nerve, and acid fruits, pickles, vinegar, &c., should be *not* used until after the operation is completed. If the gums are inflamed or turgid about the tooth, they should be freely lanced and indeed aching teeth, roots, and diseased gums should be promptly attended to as preliminary to the operation itself.

But we may be asked, if after all this trouble and the tooth is filled, it should ache, what would you do? In the first place, should the pain be but slight, we would direct counter irritation, with chloroform, tincture of arnica, camphor and laudanum, &c. If these fail, freely lance the gums and apply leeches, and finally administer a saline purgative, and if nervous restlessness supervene with diminished pain, administer six to ten grains of pulvus doveri. If these remedies should fail, take out the filling and destroy the nerve.

This leads us to the last part of the subject under consideration, which is the destruction of the nerve and the preservation of the teeth.

We rejoice to know, that however desirable living and healthy teeth may be, yet dead ones are far better than none, and that true science is claiming a victory over the forceps and mechanical dentistry. Yes! when we now stop to reflect, we can count numbers who are now rejoicing over and enjoying a fair set of natural masticating organs; who but for this victory, would be ruminating over suction plates and artificial dentures.

As preliminary to our remarks on destroying the nerve and filling the teeth, we wish to make two or three remarks. The first is, that every tooth with its pulp exposed, ought not and should not be preserved. There are crowded dentures that need relief by the judicious application of the forceps, and there are teeth having no antagonists that should be removed. In the second place, there are teeth affected with exostosis, and where wasting of the processes and disease of the gums, require that they should be extracted.

Every judicious operator will take a careful survey of the mouth before he commences to operate or decides upon what teeth should be lost.

There are cases, however, and not very seldom either, where an aching, ulcerating tooth, is of far more importance and real value, than half a dozen of sound ones that may be in the mouth.

We will illustrate this by giving a case. About a year since, a gentleman called to consult us about having out his teeth and getting a new set. A lower molar and dens-sapientia which were but little decayed, antagonized with an anterior and posterior above, which were badly decayed, the pulp exposed in one and the other discharging matter from the nerve cavity, both were frequently aching and had been condemned two years before as past recovery. The first bicuspid of the same side, superior, was decayed near the nerve and antagonized but imperfectly with the cuspid below. The latter being somewhat out of its proper place. The bicuspids on the other side above were out, and the only means of masticating on that side, was the anterior molar, which had come forward and downward, until it struck the posterior part of the posterior bicuspid below.

The cuspid and lateral incisor of this side above, were both worn down and decayed, until there nerves were exposed. Now to have taken out the three teeth with exposed nerves, and the ulcerated one, the only masticating organs left which could be of much use, would have been four front teeth above and their antagonists, virtually the loss of the diseased organs was the loss of the entire set.

Here was a fine case for the forceps and a new set of teeth; yet there was another question of far more importance than dollars and cents, which was, shall surgical or mechanical dentistry claim the pre-eminence. True science points to the former, the mechanical dentist claims the latter, and yet there can be no doubt but even such a set of teeth well preserved, are of far more real service than the best artificial dentures. We have the pleasure to know that this gentleman retains his teeth, and that they answer all the purposes of good natural organs. But for the treatment necessary to this result.

The first thing is the destruction of the nerve. The agent to accomplish this, is almost we believe, universal with the profession; arsenic, in some form or other. Some thirteen to fourteen years since, when this great remedy was being introduced as a great secret to the profession, Dr. Rostaing and ourself commenced a series of experiments to find out what it was. Arsenious acid was then used with powdered charcoal and morphia. Its black color led us to suppose it might be cobalt, and this we tried and soon found we had the agent, although in a different form from that then used. But after many experiments with various combinations of arsenious acid and other substances, we have thrown aside all other forms, and simply use the cobalt of the shops in a finely pulverized form. We believe it acts more mildly and full as efficiently as any other.

The first thing to be done, is to remove the decay so as to expose the pulp, and give room for the application in the cavity of the tooth. If the pulp is exposed and the cavity sufficient to receive the application well, no special benefit is derived by removing the decay. The object is to bring the cobalt in contact with the pulp. To accomplish this, we take a very small piece of cotton, and roll it up on the point of an instrument, touch this cotton to a cork from a phial of creosote, the cork having been wet with the creosote, this simple way of moistening the cotton avoids the too free use of the creosote. The cotton is touched to the cobalt, and the surface being moist, retains a sufficient amount of cobalt, which is then carried to the cavity and laid over the pulp, over this may be placed a cap of sheet lead, and then the cavity sealed up with adhesive wax. This should remain in the tooth some ten hours, although we often leave it in for twenty-four; usually, little if any pain is experienced, and generally the nerve is found dead. We now remove the remainder of the carious portion, and enlarge the opening into the pulp cavity. If the decay is on the approximal surface of the incisors, cuspids, or bicuspids access can be obtained pretty well, by cutting tolerably freely on the lingual face of the teeth. This we regard as the first and important step in the operation, for without easy access is made to the cavity in the fang or fangs, the whole operation is rendered difficult and uncertain. This is the point where most beginners fail.

Within the last month we have had a superior molar to treat, which had been under treatment three weeks, and yet all the remnants of the nerve and blood vessels were not removed from the nerve cavity, and hence soreness remained; and the simple reason was, that it was impossible to get into the cavities thoroughly. As soon as we cut away the tooth and removed the foreign and dead matter from the roots, soreness and discharge of matter passed off, and the tooth was soon filled.

But we do not wish to be understood as saying, that one application of cobalt will always be sufficient, and that no pain will ever be experienced to give trouble, for such is not always the case. After the first application, however, the nerve is much paralyzed, and often if not thoroughly killed, it may be caught on the end of a broach and removed entire without much pain, when this can be done we prefer to do it. We take a watch-makers broach of small size, and heat it to take out the temper, this will allow it to bend to suit the condition of the cavity, and permit it to be turned as it enters the root. As we turn the broach round and round between our fore-finger and thumb as it enters the cavity of the root, we can often feel that the nerve is being twisted on its point, some little pain may be thus given, yet not enough to cause much flinching. In withdrawing the broach the nerve will often be attached, most likely some discharge of blood or bloody matter will follow the operation, we let this pass off. Then with a few fibres of cotton wound around our broach wipe out the cavity, or throw up with a small syringe some tepid water. If the nerve cavity is very small, we enlarge it sufficiently to clear it of all extraneous matter; but usually the cavity is large enough. The question now naturally arises, shall the fang be filled at once, or must we wait to see if any discharge ensues. If the tooth is free from soreness, and after wiping out the cavity with a few fibres of cotton wrapped around the broach no blood is perceived, the cavity may at once be filled. If, however, there has been some inflammation involving the periosteum, we moisten the end of a soft thread with creosote, and having attached this to the end of our broach, carry it to the end of the fang, and pack it in firmly as a *test* temporary filling. The end of the broach will catch the fibres of the thread at its end, and by a few turns secure it sufficiently firm to be carried to the point desired; in introducing this, however, we make two or three turns of the broach as it enters the dental canal, to be withdrawn, the broach must be turned back, so as to unwind the thread. We are thus particular, because many have difficulty at first in this simple operation. After packing the thread in the canal, we cut it off and leave the end loose in the cavity of the tooth, then fill up the cavity with beeswax, and let the whole remain for one day. We then remove the wax, catch the thread on our broach and it is easily withdrawn. If this plugging up should cause pain, the thread should be withdrawn before inflammation of the periosteum takes place. If merely slight soreness by jarring of the tooth is felt, we recommend rubbing the gums around the tooth with tincture of arnica, chloroform, or camphor and laudanum. This will generally subdue the pain or tenderness in a few hours. If the tooth is free of soreness, and no bloody matter comes away with the thread, the fang is ready for filling.

Should the plugging up of the root with thread cause soreness of the teeth, pain &c., the nerve cavity should be opened by its removal; yet we re-introduce a little cotton or thread loosely, and this is moistened with creosote. The next day this is removed, and the canal thoroughly cleansed, and the thread with a little creosote introduced a little tighter. This is renewed every day, and each day made tighter and tighter, until the tooth bears it without pain, and when it is removed no flavor of pus is perceptible on the thread. During this treatment, external applications are made as counter irritants to the gums. A few days is generally sufficient for the treatment of what might usually be called bad cases.

We have, however, had a few cases which never would become quiet with a temporary filling of thread, and what is equally remarkable, they have *invariably* been satisfied when fed on gold. The first case of this kind, we had been treating for a month, several times the tooth became so sore that we threatened to take it out, yet in a few moments after the thread was removed pain ceased, and we could try it over again, lancing the gums, &c. &c., to keep down the inflammation; at last we got tired of the job, and concluded we would fill the fang, and if it ached remove the tooth. The first roll of gold introduced, gave some pain which soon passed off, and before the filling of the fang was completed, the gentleman remarked, "that feels right," and save a little tenderness on being jarred for a few days, has never troubled him since. We have had in all, perhaps, a half dozen such cases, and yet have never had to extract one of such teeth, and to our best recollection, not one has produced abscess.

We have had two or three cases, which arsenic would not kill, and we tried the pure stuff. In one case after four or five applications, and we had only got a short distance above the neck of the tooth, we waited a day or two until soreness subsided, and with a roll of foil the size of the dental canal, forced it up as near the nerve as we could without giving pain, then filled the tooth. This was five or six years since, and the gentleman has the tooth yet, and it has never given him pain.

We allude to such cases and the result, to show how successful the most undesirable cases will often terminate.

We have observed that the most of these cases, which were disposed to resist the action of arsenial preparation is, were superior cuspidati; within the last few days we have had one which gave us some trouble. This cuspid we had filled last fall, and it remained quiet until about a month or six weeks since, when it commenced to pain. We removed the filling and found that a filiment of the nerve had been exposed, and which had escaped our observation. We then applied cobalt and the lady left for St. Louis, and returned only a short time since. We found, however, that the application had not destroyed the nerve, and we made two or three applications before we could introduce our probe into the dental foramen, and then only at most a line. We then applied the cobalt direct to the nerve and sealed up tight with wax. The next day, the nerve was as quick as ever. We however, determined to carry our broach some distance up the canal, even at the risk of some considerable pain. This partially broke up the nerve, and one more application of the cobalt finished it. We this day filled the fang very satisfactorily.

There is yet a class of cases to be considered, which require a few remarks before dismissing this subject. These are cases where the pulp is already in a state of suppuration. We embrace in this class of cases, all from those in which the pulp is just undergoing decomposition, and the opening of the dental foramen, lets out bloody matter; to those which have been discharging for even years and where the closing of the canal, unless this disease is removed would, certainly ultimate in alveolar abscess.

There are many cases where the pulp has been destroyed by the action of dental caries; violent tooth-ache may have been the consequence at the time, yet after the nerve has been destroyed, the pain ceases, because the small amount of discharge which is thrown off at the apex of the fang, finds an outlet through the dental canal, close up this natural outlet, and pain and abscess is the consequence. An old method to get rid of this difficulty, was to drill into the nerve cavity under the free margin of the gum. But, however successful it may have been as a relief to the pain after such teeth had been filled, yet the disease is still there, and at best the operation is a very unscientific affair.

In the treatment of all such cases, our aim should be to remove the disease, and then save the tooth; and in the treatment of this disease, the first thing to be done is the complete removal of all irritants. We have before remarked, that we regard all foreign matter in the nerve cavity, as that which most directly keeps up this disease. In the treatment, therefore, the first thing to be done is to open perfectly the dental canal and remove all the diseased bone, and by injections of tepid water, or by some of the chlorine preparations completely *disinfect* the cavity. We say chlorine preparations, because almost any of them will answer the purpose. We use more frequently than any other, chloride of zinc in solution. The fact is, our general treatment to arrest the disease is, first wash out with the chloride of zinc where there is much discharge, and then apply the creosote as heretofore directed.

Very offensive matter may be discharged for several days after the nerve cavity is cleansed out, and if the cavity is filled too tightly with cotton or thread, pain will be the consequence. If the discharge continues copious and offensive long, a solution of six to ten grains of nitrate of silver to an ounce of water, will be found invaluable as an injection, to be used however not oftener than once in two or three days, using in the mean time every day the pure creosote on the cotton or floss silk. After the discharge has in a measure ceased, which can be told by the absence of its order on the thread or cotton which has been used, the thread should be pressed in more and more solid. If the case has been of long continuance and has required much treatment, we plug up very firmly with the thread and leave it in two or three days, and should only slight uneasiness and soreness of the tooth supervene, we leave in the thread and treat by lancing the gums, counter-irritants, &c., as before recommended.

When this soreness has subsided, we fill the nerve cavity and let it remain for two or three days and then fill the crown. We may with propriety, refer to our mode of filling the nerve cavity. We first roll on a broach, which gradually tapers to a point, a cylinder of foil about one-fourth of an inch long, as near the size of the nerve cavity as we can, this is rolled hard, and when the broach is withdrawn resembles a gold wire. This is introduced into the cavity with our plugging plyers or forceps, and then with a blunt-pointed slender instrument which can be easily introduced into the cavity, the cylinder is forced to the apex of the fang. Then another and another of like character is thus introduced and forced to its place until the cavity is completely filled. We are indebted to Dr. Clark of New Orleans, for the mode of making these cylinders. These cylinders by the way, are identical with the block which we recommended in the first series of articles which we published on the filling of teeth, excepting indeed, in the mode in which they are made. The mode of filling the crown with cylinders or blocks, which we are glad to see is attracting much attention of late, is the same as that recommended by us in our first article on this subject. The operation of filling with blocks or cylinders by some, may be considered the same as that taught by a gentleman of the name of De Ware, several years since; but his mode as far as we can learn, was to roll in small balls the foil, and that without any special system, and these were introduced with ordinary blunt and sharp pointed instruments, commencing at the bottom of the cavity and packing in ball after ball, until the cavity was filled, the arrangement of the gold in parallel lamina and inserting it as so many wedges was entirely different. Any method of introducing gold for filling requires much manipulation to become adept in, yet new beginners can certainly learn that by the use of blocks as readily as any other, and when once mastered in all its detail, we think it will not be laid aside for any other.

When we first commenced this method of filling teeth, it was in large central cavities. Its superiority soon induced us to try it in other more difficult cavities, the great difficulty, however, was, the proper instruments were not to be had. This difficulty was in a great measure overcome, by having made some five pairs of plugging forceps; long continued use has enabled us to throw aside two pair of these, and also more frequently fill up openings between the blocks, which may be made by the use of a sharp pointed instrument, with hard small sharp pointed blocks, which are forced in with serated compressing instruments.

The treatment we have given for the preservation of ulcerated teeth, may be considered very simple and often withal too tedious. Yet such is not the case, for sometimes a single tooth may beworth to an individual hundreds of dollars, and so far as simplicity is concerned, it is in strict conformity with the known pathology of the disease, and the practice based thereon by our medical brethren generally, in the treatment of like cases of ulceration.

We believe that this treatment if judiciously pursued and persisted in for a reasonable length of time, as a surgeon would for the cure of an ulcerated surface on any other part of the body, that nineteen out of twenty of such teeth can be saved, and we see no reason why not, for many, many years. There may be some constitutional habits of body, such as scrofulous diathesis, or a schirrous taint of the system, which would indicate the removal of all such teeth; yet we have not as yet met with them in our practice; that is, we have not seen injurious consequences result from such practice in the many cases we have treated; and yet, we have cases of diseased gums, showing such marked predisposition to fungus growth from dental irritation; that we unhesitatingly advise the removal of every tooth which is so far decayed as to involve the pulp organ, at least in such cases we would wait even in a front tooth, until the constitutional health and disease of the gums showed evident signs of restoration to health.

In the disease of the dental pulp, of which we have been treating, there is one stage further in advance, and which has generally been considered incurable; we mean alveolar abscess, and which has been so ably treated by Dr. Ballard of New York, that we shall merely refer to his article, and which may be found in No. 2 of volume IX of the Dental Register. We would here remark, however, that although our success in the treatment of the cases to which we have alluded, has been far greater than our most sanguine expectations could warrant; yet we still had no thought of testing it in abscess in the manner recommended by the Doctor. Still in cases of abscess, where too much of the root has not been denuded of its periosteum, we are satisfied the treatment will prove successful. The matter may cease discharging, and the abscess heal up, but that the sac at the end of the fang is removed, and the periosteum reunited to the root, is to us at least doubtful.

These articles on the treatment and filling of teeth, have occupied far more space than we anticipated, and yet we are now satisfied that a careful review of the subject would cause, us very much to extend them.

The subject of exposed and irritable nerves, and the preservation of the teeth thereafter, is, of all others, perhaps, the most important one of dental practice, and would of itself, if treated with that accuracy, which would be perfectly satisfactory to the young practitioner, make quite a volume.

